# A Treatise on the Diseases of the Chest, and on Mediate Auscultation

**Published:** 1829-04-01

**Authors:** 


A TREATISE ON THE DISEASES OF THE CHEST, AND ON MEDIATE AUSCULTATION. By *R. T. H. Laennec*, M.D. Translated from the latest French Edition, by *John Forbes*, M.D. &c. Third Edition, revised, with additional notes.

THE circumstance of a *translation* of any kind, much more the translation of a *medical work*, coming to a THIRD EDITION, is, in itself, no common phenomenon; but when we find that a large impression of this work has been sold in the short space of twelve months—of a book, too, costing not less than twenty-four shillings, we must believe that there is something more than usually attractive in such a publication: and this is certainly true of the work before us. It is of matchless excellence in its matter, and it has been excellently translated. It has, therefore, only met the success which it deserves. But we are particularly gratified, on this occasion, by viewing the rapid sale as an unequivocal proof of the great and rapidly increasing attention of the profession, in this country, to AUSCULTATION, as a means of diagnosis in thoracic diseases. We take some credit to ourselves for having been, not only the first, but the most zealous and constant advocate of this practice from the first period of its introduction, and we flatter ourselves that our labours and opinions have not been in vain, towards the furtherance of the great cause. This we may now consider as having fully triumphed; since we cannot regard the opposition still made to it, in certain quarters and by certain persons, as any thing else than what must be expected; indeed, as necessary consequences of the combined influence of their moral constitution, course of study, time of life, and civil and social relations. So far from being surprised that there are still found some disbelievers in the doctrines of Laennec, and some scoffers at the stethoscope, we are surprised that there are so few; and we congratulate ourselves on the more enlightened and liberal views and more Christian temper of the age in which we live, and the more philosophic and more tractable spirit of our own profession, which have accorded a meed to the author of *auscultation*, so different from that which attended many of the discoveries of former periods. Need we do more in reference to this difference, so flattering to our times, than call to mind the names of *Roger Bacon*, of *Harvey*, and of "the starry Galileo with his woes"? The present edition, the translator informs us, "has been carefully revised, and such alterations and improvements made in various parts of it as seemed desirable." From the new notes to this edition, we shall extract one relating to MEDIATE PERCUSSION, and another respecting a theoretical point of great importance, viz. the *cause* of the sounds produced by the action of the heart.

"An important improvement on the method of percussion was recommended some time since by M. Piorry, and has been recently fully explained in his treatise '*De*

*la Percussion Mediate.' Paris*, 1828. This improvement consists in interposing between the point of the fingers and the chest, a small plate of ivory on which the percussion is made; and from which circumstance the inventor has, in imitation of Laennec, given the name of *Mediate* Percussion to his method. The ivory plate (which has received the name of *Pleximeter* or *Plessimeter*, from the words πλήσσω, *I strike*, or πλῆξις, *percussion*, and μέτρον, *measure*,) is of a circular or ovoid shape, from an inch and a half to two inches in diameter, and about one-sixth of an inch in thickness. It has either a raised edge or rim, or projecting handles, on its upper side, to permit its being held between the finger and thumb of the left hand, while it is struck with the right. In making use of this instrument, all that seems essential is to apply it accurately, closely, and consequently parallel to the surface. As in simple percussion, the blow may be made with one or more fingers, and must be rapidly executed, with the points but not the *nails* of the fingers: on this account the nails must be kept short. The pleximeter may be applied immediately on the skin or over some portion of the clothes; and, as in the case of the stethoscope, it is necessary on some parts to interpose a small pledget of lint or soft linen, to ensure its accurate apposition.

"The following are the principal advantages which mediate percussion has over direct or simple percussion :—1. Owing to the diffusion of the impulse of the blow over a much larger space, and the impulse necessary to elicit sound being itself less, percussion by means of the pleximeter can be applied on parts that are inflamed or painful, as in the case of a blister or issue, in eruptions, &c. &c.; for the same reasons, there is no fear of painfully affecting or injuring by the concussion communicated to the walls of the chest, the diseased parts within, a circumstance not infrequently observed in cases of pleurisy both acute and chronic. 2. It permits a more extensive application of the process; as, by means of the pleximeter, we can percuss every part of the chest, on an intercostal space or superficial muscle for instance, as well as a rib. 3. In using the pleximeter, we need not be solicitous, as in the case of direct percussion, of selecting precisely similar points on the two sides of the chest, for comparison. 4. Mediate percussion is easier than the old method; as having in the pleximeter always a perfectly level surface, we can with much more facility impinge our fingers with the requisite perpendicularity, than we can do on the uneven surface of the chest. 5. It is also more simple than immediate percussion; as we uniformly employ the same process on every point of the chest; whereas in the method of Avenbrugger, we must sometimes strike across the ribs and sometimes parallel with them. 6. Mediate percussion is more precise and minute in pointing out the limits of the diseased part, as the sound elicited from the pleximeter is derived exclusively from the point struck. We may have a familiar illustration of this in placing the instrument (or any thin flat solid) upon the side of the face, so as partly to rest on the inflated cheek, and partly on the cheek-bone: in tapping the plate in this position, we shall find that the difference of the subjacent parts is accurately pointed out by the difference of sound. In like manner, in the case of pleuritic effusion or hydrothorax, we can trace with the utmost precision the upper level of the fluid within the chest. For these and other reasons it cannot be doubted that *Mediate Percussion* enables us to obviate several of the difficulties and imperfections of the old method, and that it is therefore well entitled to the attention of practitioners. It has, moreover, the great and peculiar merit of being applicable to the diseases of the abdomen. For ample details on every point relating to this method, I refer the reader to M. Piorry's very valuable work. Different things have been used as *pleximeters*, and, among others, the horn cap, which is now commonly affixed to the auricular extremity of the stethoscope. M. Piorry objects to this, on account of the liability of horn to warp, and also on account of the perforation in its centre. Dr. Williams, however, seems to consider this last as no objection, but recommends the inner surface of the cap to be lined with soft leather, to prevent the clacking noise produced by the impulsion of the fingers. (*Rational Exposition*, p. 22.) My own experience is against the use of the perforated pleximeter; exclusively of an objection I have to the cap of the stethoscope being so made as to be easily removed. With the view of making the instrument conveniently portable, one variety of M.

Piorry's pleximeter might be made to fit to the pectoral extremity of the common stethoscope." 24.

It is known to all auscultators, that the FIRST *of the two immediately successive sounds* heard in exploring the heart, is ascribed by Laennec to the contraction of the *ventricles*, and the second to the contraction of the *auricles;* while the *interval of silence* after the second sound is, of course, considered by him as a pause between the termination of the contraction of the auricles and the commencement of that of the ventricles. On this explanation, Dr. Forbes has the following note.

"In a memoir just published in vol. iii. of the Transactions of the Med. Chir. Soc. of Edinburgh, p. 205, Mr. Turner has called in question the accuracy of this account of the motions of the heart, and, consequently, the accuracy of the relation of the sounds heard by the stethoscope, to these motions, as described by Laennec. Mr. Turner has shewn the general opinion of physiologists to be, that the contraction of the auricles *immediately precedes* that of the ventricles, and that the interval of repose is after the contraction of the *ventricles,* and not after that of the auricles, as was the opinion of our author. Mr. Turner states that this opinion is further supported by his own personal observation of the phenomena of the circulation in men and animals; and he comes to the conclusion that the last of the two sounds, accompanying the motion of the heart, is produced by some other cause than the contraction of the auricles. The systole of the auricles he considers either as productive of no sound, or of a sound which is blended with that of the ventricles. He conjectures that the second sound may 'be accounted for by the impulse occasioned by the falling back on the pericardium of the relaxed heart in its diastole, after it has been elevated or moved from its place in the systole.' I regret that the publication of Mr. Turner's paper, only just as the present edition is going to press, prevents my investigating this point which must be considered as of the very first importance in a practical point of view. For, as Mr. T. justly observes, if his views be correct, 'it is obvious that an error pervades the otherwise valuable descriptions given by Laennec of the actions and diseases of the heart.' I may here add, that it has on several occasions occurred to me, in practice, to find it very difficult to reconcile the explanation of the action of the heart, as given by Laennec, with the phenomena before me; although, at present, I cannot think the explanation of Mr. Turner the true one. I recommend this subject to the especial notice of Dr. Williams." 559.

We cannot occupy more of our space at present with further extracts from this most valuable work; but we cannot conclude without recommending it to our readers in the strongest terms, and *for the third time*, but, we hope, not the last, as the most comprehensive and valuable work which has ever been published on the pathology and diagnosis of diseases of the chest.

